# Editorial: Host-parasite-vector interactions: Invasion and persistence

**DOI:** 10.3389/fcimb.2022.1087131

**Published:** 2022-11-22

**Authors:** Devki Nandan

**Affiliations:** Department of Medicine, University of British Columbia, Vancouver, BC, Canada

**Keywords:** host-pathogen interactions, intracellular pathogenesis, *Leishmania*, leishmaniasis, protozoan parasites

This Research Topic, *Frontiers in Host-Parasite-Vector Interactions: Invasion and Persistence*, received six articles, including four original research studies and two reviews. All four original articles and one review paper relate to the intracellular protozoan parasite *Leishmania*, its interaction with the host, and resulting pathogenesis. The other review article updates our understanding of the biochemical and physiological mechanism of gametogenesis in *Plasmodium*, which could be targeted to control parasite and malaria transmission, and highlights gaps that still exist within our understanding of the process of gametogenesis. Together, these articles are related to protozoan parasites causing human diseases. This is not surprising as parasitic infections are responsible for severe mortality and morbidity worldwide, particularly in developing countries. In fact, millions of lives are lost due to parasitic infections each year, and most mortality and morbidity are due to protozoan infections. Most protozoan infections are caused by *Leishmania* spp., *Plasmodium* spp., and *Trypanosoma* spp. These pathogens can establish persistent and sometimes long-term infections. Several of these intracellular pathogens have developed diverse immune escape strategies and can overcome immune responses by residing and replicating inside host immune cells, primarily mononuclear phagocytic macrophages.

Although macrophages are armed with potent anti-infection properties, there are certain microbial pathogens, including several parasites like *Leishmania* spp. *and Trypanosoma* spp., that are capable of actively infecting these hostile cells. To establish infection, these intracellular parasites must first find ways to enter their host cells. For example, some pathogens (*Leishmania*, *Trypanosoma*) exploit phagocytic pathways by engaging specific phagocytic receptors. Once inside, these pathogens must employ clever strategies to survive, grow, and replicate in a hostile intracellular environment. Such strategies include deactivating the host cells by modulating host response by subverting intracellular signal transduction pathways of activation. A deeper understanding of the immune evasion mechanisms used by these devastating protozoan parasites can pave the way to develop targeted therapies to treat diseases caused by these parasites. Studies presented in this special issue of “Frontiers in Cellular and Infection Microbiology” serve as a significant step in the right direction by revealing the complex interface between parasites and their hosts.

Singh et al. showed that upregulation of the expression of CD300a, an immune inhibitory receptor, on antigen-presenting phagocytic cells resulted in the inhibition of their anti-microbial effector functions. Abrogation of CD300a signals in *Leishmania-*infected mice helped in the early clearance of parasites from their visceral organs and implicated CD300a as an important determinant of phagocytic host-cell function and T cell differentiation against *Leishmania* antigens.

Recent understanding of the role of *Leishmania* secretory exosomes/extracellular vesicles on leishmaniasis unlocked a new area of potential and intricacy regarding our understanding of the disease. In this regard, Shokouhy et al. isolated extracellular vesicles (EVs) from non-pathogenic *L. tarentolae* and functionally tested these vesicles in mice infected with *L. major* for the production of cytokines and pathogen survival. Upon prior treatment with *L. tarentolae* EVs (tEVs) in *L. major-*infected human monocytic cells (THP-1), there was a significant increase in the production of proinflammatory cytokines like IFN-γ, TNF-α, and IL-1β, but not anti-inflammatory cytokines like IL-6 when compared to cells treated with *L. major* EVs prior to the infection. Excitingly, they also observed a significant decrease in parasite survival in tEV-treated *Leishmania*-infected macrophages and advocated tEVs as a potential drug delivery platform for parasitic infections.

In order to understand the skin-parasite landscape, Doehl et al. showed that initial inoculation of *Leishmania donovani* parasites in the skin led to the clustering of myeloid cells resembling the shape of innate granulomas, followed by further infection in the newly recruited cells, eventually translating to self-propagating networks of patch clusters. This presents a novel perspective on the behaviour of phagocytic cells and the skin-parasite landscape following infection. Lastly, Sghaier et al. examined the healed lesions caused by *Leishmania major* infection in humans for the persistence of parasites. This study involved biopsies of cutaneous scars from 53 volunteers cured of cutaneous leishmaniasis. These tissue samples were investigated for the presence of residual parasites using multiple methods. Unexpectedly, their investigation did not find evidence of residual pathogens. This situation contrasts with other *Leishmania* species causing chronic, diffuse forms of leishmaniasis where parasites persist in healed lesions. It will be of interest to extend this observation to other anatomic sites like lymph nodes. Such findings could be valuable in guiding the design of effective vaccines against cutaneous leishmaniasis in the near future.

The review article by Al-Khalaifah focuses on major molecular factors related to *Leishmania* pathogenicity. Most of these molecular factors are also known as “virulence factors” and are considered ideal targets for designing small molecule inhibitors to attenuate the survival of *Leishmania* spp. He also touches on metabolic changes of host macrophages in relation to *Leishmania* infection and survival. It was interesting to note that some infections dampen the immune response of their mammalian hosts by the depletion of amino acids that are essential to immunological processes. Thus, it is becoming increasingly clear that *Leishmania* manipulates host metabolic fluxes to evade host immunity, resulting in prolonged *Leishmania* survival and pathogenicity. This paper also highlights the importance of further research into *leishmanial* virulence factors, and how this could aid in the development of novel therapeutics to treat disease by providing better knowledge of disease pathogenesis and etiology.

A review article by Dash et al. entitled “*Gametogenesis in Plasmodium: Delving Deeper to Connect the Dots*” summarizes potential strategies to control the transmission of malaria. One such strategy is blocking the gametocyte-gamete transition, thus potentially restricting the successful transmission and progression of the disease. This can then be exploited for designing transmission-interfering strategies. As a result, Dash et al.’s review mainly focuses on summarizing the current understanding of the biochemical and physiological mechanisms involved in gametogenesis in the malaria parasite *Plasmodium*, which could be targeted to control parasite and malaria transmission. Interesting questions were raised regarding gametogenesis biology in *Plasmodium*, and are worth investigating for a better understanding of the process of gametogenesis.

Taken together, the six papers of our Research Topic provide a valuable addition to the field of host-pathogen interactions. This includes a number of exciting new scientific findings regarding emerging evasion strategies used by intracellular pathogens like *Leishmania*. We hope these contributions will help investigators in the field of host-pathogen interactions and intracellular pathogenesis in their continuous scientific pursuit to understand the molecular mechanism of the pathogenesis of intracellular protozoan pathogens. This could pave the way to develop effective clinical therapeutics to treat human diseases caused by these protozoan pathogens.

## Author contributions

The author confirms being the sole contributor of this work and has approved it for publication.

## Conflict of interest

The author declares that the research was conducted in the absence of any commercial or financial relationships that could be construed as a potential conflict of interest.

## Publisher’s note

All claims expressed in this article are solely those of the authors and do not necessarily represent those of their affiliated organizations, or those of the publisher, the editors and the reviewers. Any product that may be evaluated in this article, or claim that may be made by its manufacturer, is not guaranteed or endorsed by the publisher.

